# Using recruitment data instead of national population ethnicity proportions in clinical trial preparation may introduce bias

**DOI:** 10.1177/01410768241299529

**Published:** 2024-11-19

**Authors:** H Logan Ellis, J Smith, AM Murtagh, M Al-Agil, MB Whyte

**Affiliations:** 1 Department of Diabetes, King’s College Hospital NHS Foundation Trust, London, SE5 9RS, UK; 2 Department of Critical Care, King’s College Hospital NHS Foundation Trust, London, SE5 9RS, UK; 3 Department of Research & Innovation, King’s College Hospital NHS Foundation Trust, London, SE5 9RS, UK; 4 Kings College Hospital, King's College Hospital NHS Foundation Trust, London, SE5 9RS, UK Email: m.b.whyte@surrey.ac.uk

The systematic review by Kayani et al.^1^ of European and North American data suggested the under-representation of ethnic minorities in research, using recruitment data versus national population ethnicity proportions as the denominator. However, this may introduce bias, as national ethnicity data may not be representative of the population in the environs of a research centre and/or may not accurately reflect the ethnicity composition of specific disease areas, such as prostate cancer and type 2 diabetes, which are disproportionately more common among Black populations. We have examined ethnicity participation in research, per ethnicity group, from a large, urban teaching hospital and compared the proportions directly with the population utilising the outpatient and inpatient services of the same hospital Trust. We utilised Cogstack, a natural language processing tool, to analyse clinical data in electronic health records (EHRs) using the MedCAT library. We examined patient records for clinical research participation indicators, including textual notes and research consent forms, identifying keywords like ‘clinical trial’, ‘consent’ and ‘study number’. Patients with these keywords were linked to ethnicity data in the EHRs. We compared clinical trial participation (1 January 2000–10 February 2023) by ethnicity to the aggregate of overall healthcare user demographics at King’s College NHS Trust from the last 25 years. Ethnicity categories were those used by NHS Digital, and are self-reported.

From 1.5 million patients, 1204 had research entries. White British ethnicity was most common among research participants (49.6%) as well as the overall patient population (43.2%). Black African patients were 6.2% of the overall population but 4.5% among research participants; Black British were 4.7% of the overall population but 3.4% among research participants; ‘Other ethnic group’ were 3.4% of the overall population but 2.6% among research participants. Notable variations included Indian/British Indian (1.4% overall; 1.9% in research) and ‘white’ (3.6% overall; 0.7% in research).

Even when using the proportions accessing outpatient and inpatient services as a denominator, ethnic minority groups appear underrepresented in clinical research. Further work is needed to understand ethnic disparities in clinical trial participation to advance healthcare equity and personalised medicine.

## Declarations

### Guarantor

MBW.

### Provenance

Not commissioned; peer reviewed by Ash Routen.

### ORCID iD

MB Whyte https://orcid.org/0000-0002-2897-2026
